# Effect of photobiomodulation on pain relief and functional improvement in fractures: a systematic review and meta-analysis

**DOI:** 10.1080/07853890.2026.2667061

**Published:** 2026-05-04

**Authors:** Weifeng Wang, Ruijuan Xiu, Xiaohua Zhao, Xueling Qiu, Lu Tang

**Affiliations:** ^a^ Department of Stomatology, the 960th hospital of People’s Liberation Army of China (PLA), Jinan, China; ^b^ School of Nursing, Shandong Second Medical University, Weifang, China; ^c^ Sick and Wounded Management Section, the 960th hospital of People’s Liberation Army of China (PLA), Jinan, China; ^d^ School of Nursing, Shandong First Medical University, Taian, China

**Keywords:** Photobiomodulation, low-level laser therapy, fracture, pain, Rehabilitation

## Abstract

**Introduction:**

Fractures, the most common type of trauma, can cause considerable distress to patients. Pain can not only affect the comfort of fracture patients but also delay their participation in rehabilitation training. Photobiomodulation (PBM) has been associated with pain reduction and the promotion of tissue healing. This systematic review and meta-analysis aimed to evaluate the efficacy of PBM in reducing pain and promoting rehabilitation in patients with fractures.

**Methods:**

This study was registered on PROSPERO (CRD42024591373). We systematically searched PubMed, EMBASE, the Cochrane Library and Web of Science for RCTs that investigated PBM in fractures as of August 2025. The primary outcome was the pain score. The secondary outcomes included functional and healing.

**Result:**

Finally, 12 and 9 studies were ultimately included in the systematic review and meta-analysis, respectively. The pooled analysis showed that the one-week pain score was lower in the PBM group than in the placebo group (MD −0.74, 95% CI −1.00, −0.47, *p* < 0.0001, I^2^ = 0%). Subgroup analysis showed that the difference between the two groups was statistically significant regardless of fracture site or acupoint irradiation. Changes in pain scores were statistically significant in both groups at different wavelength combinations. The improvement in grip strength at 4 weeks was significantly greater in PBM than in placebo (MD 5.03, 95% CI 4.29, 5.78; *p* < 0.0001; I^2^ = 0%). There were no significant differences in pain and functional scores at 4–26 weeks. Bone healing did not show differences between the two groups. No side effects reported.

**Conclusion:**

PBM appears to relieve short-term pain in fractures and improve grip strength in patients with upper limb fractures, but does not show significant long-term benefits. Evidence for mandibular functional recovery and bone healing remains inconsistent. Future studies should determine therapeutic parameters and their impact on bone healing and long-term functional outcomes across fracture types.

## KEY MESSAGES

Photobiomodulation can reduce short-term pain in patients with fractures (one week after injury), but the current evidence shows no significant long-term effects.

Photobiomodulation is helpful for grip strength improvement at four weeks in patients with upper limb fractures, and the function of mandibular fractures is still controversial.

In the future, it is necessary to study the influence of parameters and irradiation on the pain and function of different types of fractures.

## Introduction

As one of the largest systems in the human body, bones allow for a variety of complex movements while providing stability. It plays a crucial role in maintaining the function of the blood system and muscle system [1,2]. Fracture is a common traumatic disease related to sports and accidents. The management of bone injury usually includes fixation, surgery, rehabilitation, etc. Despite the regenerative capacity of bone, the healing of some fractures requires a lengthy time as well as repeated surgical interventions [3]. Injury and bone rehabilitation are often accompanied by severe acute pain, which gradually decreases as the bone heals [4,5].

In addition, early rehabilitation after fracture surgery is very important and involves preventing muscle atrophy and joint stiffness, promoting bone healing and improving blood circulation. However, many patients cannot fully participate in early rehabilitation treatment. Nonsteroidal anti-inflammatory drugs (NSAIDs) are the first-line option for managing pain after fracture, but long-term use may inhibit bone healing [6]. Although opioids have significant analgesic effects, their use is limited by adverse reactions such as addiction, constipation and respiratory depression [7]. In recent years, more and more emphasis has been placed on restricting the use of opioids in clinical practice. Acetaminophen and muscle relaxants as adjuvant drugs also have limitations such as hepatotoxicity or sedation [8,9]. In this context, photobiomodulatory therapy, as a non-invasive, non-thermal physical therapy with few adverse effects, has attracted attention in the field of fracture rehabilitation in recent years. For fracture patients, conventional drug analgesia is essential. PBM can be used as an auxiliary choice in multimodal analgesia and rehabilitation programs, which can reduce the dosage of drugs and adverse reactions while assisting analgesia.

Photobiomodulation, also known as low-level laser therapy, is a physical therapy method. Biological tissues are irradiated with a laser of a specific wavelength to promote cell function recovery and tissue repair with a nonthermal effect [10]. In recent years, animal experiments and human experiments have shown that photobiomodulation therapy can reduce pain, control the inflammatory response, and accelerate damage repair through various mechanisms, such as regulating the inflammatory response, promoting microcirculation, and enhancing cell metabolism [11].

From a molecular perspective, low-level laser light interacts with specific molecular components of biological tissues to modulate their cellular function. The mechanism of photobiomodulation involves excitation of the mitochondrial respiratory chain, which is one of the most critical cellular responses. Cytochrome C oxidase, located in Unit IV of the mitochondrial respiratory chain, absorbs red light or near-infrared light [12], leading to increased enzyme activity [13] and increased mitochondrial respiration and adenosine triphosphate (ATP) synthesis levels [3,14]. In addition, it can stimulate the activities of various molecules, such as nitric oxide, calcium ions, reactive oxygen species and numerous other signaling molecules [15], including cytokines involved in cell proliferation, survival, tissue repair and healing [16].

Several studies, including systematic reviews, have reported the effects of photobiomodulation on pain, inflammation, and bone repair in patients with orthopedic diseases [17–19]. A systematic review by Neto FCJ et al. evaluated the effectiveness and safety of photobiomodulation in the treatment of fractures [20]. However, they included only two randomized controlled trials, and the evaluation of the quality of evidence was low. In addition, several randomized controlled trials of photobiomodulation in fractures have been reported in recent years. In view of this, this systematic review aims to evaluate the efficacy and safety of PBM in the treatment of fractures on the basis of existing studies to provide evidence for its clinical application.

## Methods

The systematic review and meta-analysis were developed according to the preferred reporting items for Systematic Reviews and Meta-Analyses (PRISMA) guidelines [21]. The protocol was registered in the PROSPERO database with the identifier CRD42024591373. We have not published the relevant protocol for this study. This study collected data only from published studies, so no public participation or ethical approval was required for the study design or process.

### Search strategy

To identify relevant articles, both authors searched the following databases separately: PubMed, EMBASE, the Cochrane Library and Web of Science. We conducted a systematic search using MeSH terms and appropriate corresponding keywords. The search strategy is shown in Table 1. No restrictions were imposed on the study design, date or language. We conducted a manual search of the list of references for the review and included studies to identify reports that may be relevant but were missed through electronic searches. All the databases were searched on the same day (August 1, 2025), and all the identified studies were included in the title and abstract screening.

**Table 1.** Search strategy.

ORDER	STRATEGY
#1	Search: “Photobiomodulation”
#2	Search: “Laser”
#3	Search: “Photobiomodulation Therapy”
#4	Search: “Low Level Light Therapy”
#5	Search: “Low-level laser”
#6	Search: “Low-Level Light Therapies”
#7	Search: “Biostimulation, Laser”
#8	Search: “Laser Irradiation, Low-Power”
#9	Search: “Laser Phototherapy”
#10	Search: “Laser Therapy, Low-Power”
#11	Search: “Low-Power Laser Irradiation”
#12	Search: “Low-Power Laser Therapy”
#13	#1OR #2 OR #3 OR #4 OR #5 OR #6 OR #7 OR #8 OR #9 OR #10 OR #11 OR #12
#14	Search: “Fracture”
#15	Search: “Bone fractures”
#16	Search: “Broken Bones”
#17	#14 OR #15 OR #16
#18	Search: “Pain”
#19	Search: “Analgesia”
#20	Search: “Suffering, Physical”
#21	Search: “Ache”
#22	Search: “Aches”
#23	Search: “Function”
#24	Search: “Functional recovery”
#25	Search: “Functional outcome”
#26	Search: “Rehabilitation”
#27	Search: “Disability”
#28	Search: “Healing”
#29	Search: “Fracture Healing”
#30	#18 OR #19 OR #20 OR #21 OR #22 OR#23 OR#24 OR #25 OR #26 OR #27 OR #28 OR #29
#31	#13 AND #17 AND #30

### Eligibility criteria

Studies that met the following criteria were included in this review.

#### Study type

We included randomized controlled trials that followed the PICOS framework [22].

#### Participants

The study was conducted in patients with fractures > 18 years of age.

#### Intervention

The application of photobiomodulation (or low-level laser) therapy to fracture patients aims to alleviate patient pain and promote fracture rehabilitation.

#### Comparators

The control group was given a placebo or conventional therapy.

#### Outcomes

The outcome indicators for the included trials must show the intensity of the patient’s pain in the form of a score, including the VAS and NRS. The secondary outcomes may include the following: bone healing, fracture rehabilitation, and side effects.

### Screening and data extraction

Two reviewers (WFW and XLQ) independently screened titles and abstracts selected from the search *via* Endnote X9 (Thomson Scientific, USA), removed duplicate studies and identified studies on the basis of the inclusion and exclusion criteria. After that, the reviewers assess the eligibility of the full-text content to determine if it is ultimately included. When multiple publications appeared in the same study population, the most recent report with the largest sample size and outcome measures meeting the eligibility criteria was selected. The screening process and reasons for exclusion are shown in the PRISMA flowchart. The data were extracted independently by two reviewers (WFW and XLQ). The following data were extracted: first author, year of publication, year of baseline study, country, intervention, control, blinding, race of subjects, number, sex, age, parameters of the therapeutic device, quantitative outcomes, measurement instruments, and narrative summary of outcomes (e.g. side effects). We contacted the study author by e-mail if key data or information was missing from the article. Any disagreements or uncertainties were discussed by the two reviewers until a consensus was reached. If necessary, the results were discussed with a third researcher (XCJ).

### Assessment of risk of bias and the quality of evidence

This study’s risk of bias was independently assessed by two researchers (WFW and XLQ) *via* the Cochrane Collaboration Risk of Bias 2 (RoB 2) tool to assess bias arising from the randomization, bias due to deviation from established interventions, bias from missing outcome data, bias from outcome measures, and bias from selective reporting. Each domain was classified as ‘low risk’ “some concern,” or “high risk,” and each trial’s overall risk of bias follows its highest risk of bias.

The certainty of the evidence was evaluated *via* the Grading of Recommendations Assessment, Development, and Evaluation (GRADE) by two researchers (WFW and XLQ) [22]. The scoring method considers the limitations of the study, including the risk of study bias, directness, consistency, precision and publication bias, to evaluate the certainty of the combined effect size. The quality of evidence was determined at one of the following four levels: high, moderate, low, and very low.

### Outcomes

#### Primary outcome

The primary outcome was the patient’s pain score, defined as the degree of pain the patient experienced during the fracture. When pain was assessed at more than one time point, we calculated the combined effect size on the basis of the combination of studies at different time points. In this review, the main outcomes were measured by four scales:

1. Visual Analog Scale (VAS) [23].
2. Numerical Rating Scale (NRS): The NRS is a simple tool used to assess pain intensity on a scale of 0–10 points, where 0 = no pain and 10 = worst possible pain.
3. McGill Pain Questionnaire (MPQ): A multidimensional comprehensive pain evaluation scale.

If the number of included studies was insufficient to calculate the combined effect size, we included them only in systematic reviews.

#### Secondary outcome

The secondary outcomes included functional recovery, fracture healing, and analgesic consumption. Functional recovery was evaluated by grip strength for patients with upper limb fractures (kg) and the maximum opening distance for patients with mandibular fractures (mm). The evaluation method of bone healing is a radiographic description. Consumption of analgesic drugs was evaluated by occurrence or absence. Patient-Rated Wrist and Hand Evaluation (PRWE): The PRWE is a 15-item questionnaire allowing patients to rate their levels of wrist pain and disability.

### Data analysis

We used Review Manager software (REVMAN v5.3 Cochrane Collaboration) for data analysis. The combined effect size of the quantitative data was expressed as Standard mean difference (SMD) or weighted mean difference (MD) with 95% confidence interval (CI) and the risk ratio (RR) with 95% confidence interval (CI) for the dichotomous outcome measures. The results of the meta-analysis are presented as a forest plot. Q statistic (*p* < 0.1 indicates significance) and I^2^ test was used to analyze the heterogeneity of the included studies.I^2^ values of 0-30%, 30%-50%, 50%-70% and 70–100% will be respectively considered low, moderate, considerable and substantial heterogeneity. If *p* *<* 0.1 and I^2^ value is < 50%, the included studies have low heterogeneity, using the fixed-effect model; If *p* ≤ 0.1and I^2^ values are ≥ 50%,the heterogeneity of the included studies is high and the random effects model will be used. When heterogeneity was high, the sources of heterogeneity were explored *via* subgroup analysis. Potential sources of heterogeneity were explored *via* sensitivity analyses (sequentially excluding one study observing changes in pooled effect sizes), and the robustness of the results was assessed. Publication bias was assessed by visually inspecting a funnel plot when ten or more trials were available.

## Results

### Search and selection

**Figure 1.**
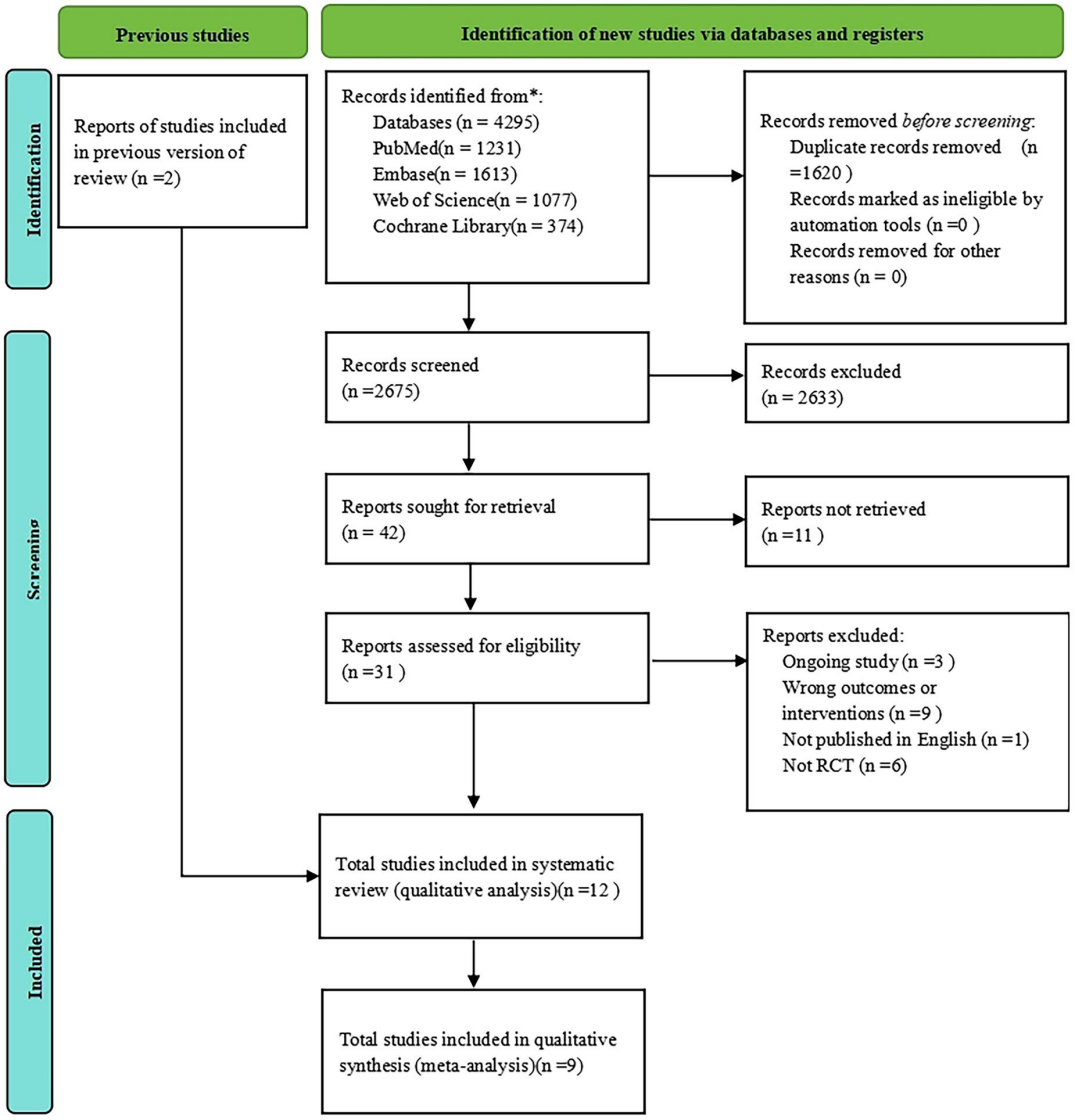
Flow chart of study selection.

We retrieved 4295 results from four databases with 1620 replicate studies. After the titles and abstracts were reviewed, 31 full articles were read to identify the retained studies. Finally, a total of 12 studies were included in the systematic review [24–35]. However, two of these studies had outcome measures presented as statistical graphs [28,34], and we were unable to obtain specific data to combine effect size. Additionally, one study used a unique pain scale that could not combine effect sizes [25]. Therefore, 9 studies were ultimately included in the meta-analysis (Figure 1).

### Study identification

The included studies were published between 2014 and 2025,with a total of 476 patients. All studies reported basic characteristics of patients (Table 1), and there was no statistical difference in gender or age. A total of 257 patients were included in the intervention group, and 219 patients were included in the control arm. The included trials were conducted in five countries: 2 in Norway [32,33], 2 in China [26,29], 4 in Brazil [25,27,28,34], 1 in Mexico [30], and 3 in Iran [24,31,35]. Two studies were conducted by the same team [32,33]. All included studies were randomized controlled trials, with 1 trial containing multiple groups [29]: traditional acupuncture (TA), laser acupuncture (LA) and sham laser acupuncture (SLA), in which the TA and LA groups were stimulated at the same acupuncture points with filiform needles and lasers, respectively. In 3 studies, the laser irradiated specific acupuncture points [24,29,30], and in 9 studies, the irradiation points were determined according to the location of the fracture [25–28,31–35]. The control group of one trial did not receive any treatment [25]. The control group in 11 trials was irradiated with a sham laser at the same site as the intervention group was [24,26–29,31–35]. The fracture sites included the upper and lower limbs [25,26,30–33], ribs [29], and mandible [27,28,34,35]. The characteristics of the included studies are shown in Table 2.

**Table 2.** Characteristics of included randomized controlled trials.

Study ID	Country	Participants			Type of fracture	Laser Type	Wavelength	Energy density	Potency	Control	Irradiation time	Irradiation site	Treatment time and follow-up	Outcomes
		No	Gender	Age										
		L/C	M/F	years										
**Nesioonpour,S, 2014**	**Iran**	**27/27**	**None**	**L:25.05** **±2.68** **C:24.61** **±2.76**	**Tibial Fracture** **(Lower limb)**	**Combination of two lasers:** **GaAlAs** **GaAlInP**	**1- 808 nm** **2- 650 nm**	**1- 6 J/cm^2^** **2- 3 J/cm^2^**	**300mW**	**Placebo laser**	**50sec** **per point**	**Fracture region** **(Contact skin)**	**Sessions:Only on the first postoperative day.** **Follow-up:2, 4, 8, 12, and 24 h after surgery**	**1.Pain (VAS)** **2.Duration of surgery** **3.Duration of anesthesia**
**Chang, W,** **2014**	**China**	**25/25**	**L:16/9** **C:13/12**	**L:33.64** **±7.82** **C:30.56** **±9.61**	**Closed bone fractures** **(Upper limb)**	**NR**	**830nm**	**9.7 J/cm^2^**	**60mW**	**Placebo laser**	**600sec** **per point**	**Fracture region** **(Contact skin)**	**Sessions:Once a day, 5 days a week, lasted 2 weeks.** **Follow-up:Before the treatment, after the treatment,a 2-Week Follow-Up**	**1.Pain (VAS)** **2.Function(Quick DASH)** **3.Ggrip strength** **4.Radiographic:Absent FL/Detectable CB**
**Acosta-Olivo, C.2017**	**Mexico**	**13/13**	**L:5/8** **C:4/9**	**L:59.2** **±14.7** **C:53.2** **±9.7**	**Distal radius fracture(Upper limb)**	**NR**	**980 nm**	**NR**	**50mW**	**Placebo laser and** **exercise**	**30sec** **per point**	**Acupoints**	**Sessions:A total of 10 sessions with a frequency of 3 times a week.** **Follow-up:2 weeek, 3, 4, and 6 week after surgery.**	**1.Pain (VAS)** **2.PRWE** **3.Wrist Mobility** **4.Adverse Events**
**Lauriti, 2018**	**Brazil**	**6/6**	**12/0**	**Mean** **age:34.5**	**Mandibular fractures** **(facial)**	**GaAlAs**	**659.93 nm**	**21.6J/cm^2^**	**108mW**	**Placebo laser**	**15sec** **per point**	**Acupoints**	**Sessions:Immediately during the first postoperative week and three treatments per week after 7, 14, 30, and 60 days.** **Follow-up:7, 14, 21, 30, and 60 days after surgery.**	**1.Pain (VAS)** **2.Mandibular dynamics:** **Mouth openning,Right** **and left movements,** **Protrusion.** **3.Facial swelling**
**Dos Santos,** **2021**	**Brazil**	**7/7**	**L:6/1** **C:4/3**	**L:51.84** **±17.31** **C:45.58** **±13.11**	**Mandibular fractures** **(facial)**	**NR**	**808 ± 10nm**	**8J/cm^2^**	**100mW ± 20%**	**Placebo laser**	**120sec** **per point**	**Fracture region** **(Contact skin)**	**Sessions:24 h and 48 h after surgery.** **Follow-up:One week after discharge** **,weekly for 4 weeks.**	**1.Pain (VAS)** **2.Mandibular mobility** **3.Facial sensitivity**
**Saebø, H,** **2021**	**Norway**	**23/23**	**L:19/8** **C:20/6**	**L:52.44** **±13.98** **C:51.08** **±16.01**	**Distal radius fracture** **(Upper limb)**	**GaAs**	**904nm**	**Total Dose 6.6 J**	**60mW**	**Placebo laser**	**60sec** **per point**	**Fracture region** **(Contact skin)**	**Sessions:9 times within 3 weeks.** **Follow-up:Baseline (1–3 days after injury),week 3 post injury,4, 8, 12, and 26 weeks after trauma.**	**1.PRWE** **2.AROM** **3.Grip strength** **4.pain pressure** **threshold**
**Saebø, H,** **2022**	**Norway**	**23/27**	**L:19/4** **C:24/3**	**L:59 ± 14** **C:57 ± 14**	**Distal radius fracture** **(Upper limb)**	**GaAs**	**904nm**	**Total Dose 7.2 J**	**60mW**	**Placebo laser**	**20sec** **per point**	**Fracture region** **(Contact skin)**	**Sessions:9 times within 3 weeks.** **Follow-up:4(baseline = cast removal), 7, 8, 12, and 26 weeks after DRF injury.**	**1.PRWE** **2.Night pain** **3. Analgesic medication**
**Liu,Chun-Ting, 2022**	**China**	**L:37** **A:37** **P:35**	**L:22/15** **A:27/10** **P:20/15**	**L:26.15** **±5.28** **A:26.38** **±5.33** **P:24.27** **±3.46**	**Traumatic rib fracture**	**GaAlAs**	**810nm**	**12.5 J/cm^2^**	**150mW**	**A:filiform needles** **P:Placebo laser**	**5sec** **per point**	**Acupoints**	**Sessions:Once daily for three consecutive days after the day of enrollment.** **Follow-up:Days 1 to 3 after treatment.**	**1.Pain(NRS).** **2.SMI** **3.Stress response** **4.Use of medications** **5.Complications** **6.Length of hospital stay**
**Bandari,** **2022**	**Iran**	**20/20**	**L:5/15** **C:5/15**	**L:28.95** **±5.23** **C:31.2** **±5.69**	**Mandibular fractures** **(facial)**	**GaAlAs**	**808nm**	**100 J/cm^2^**	**100mW**	**Placebo laser**	**20sec** **per point**	**Acupoints**	**Sessions:Seven sessions were held right after the surgery and the following days until the opening MMF after one week.** **Follow-up:One week after treatment.**	**1.Pain (VAS)** **2.The level of jaw movement.(using ruler)**
**Bonfim,D.S, 2024**	**Brazil**	**10/10**	**Male 100%**	**L:31.1** **±13.43** **C:37.3** **±14.26**	**Upper limb fracture or Lower limb fracture**	**NR**	**780nm**	**10 J/cm^2^**	**40mW**	**None**	**10sec** **per point**	**Fracture region** **(Contact skin)**	**Sessions:Twice a week for 60 days, totaling 16 sessions per patient.** **Follow-up:Immediate postoperative period, the 30^th^ day of treatment,the 60^th^day of treatment.**	**1.Pain(MPQ)** **2.Analysis of the digital radiographic examinations** **3.Dosage of inflammatory cytokines**
**Pavelski MD** **2024**	**Brazil**	**L:13** **P:10**	**Male 70%**	**Mean** **age:33.1**	**Mandibular fractures** **(zygomatic)**	**AsGaAl**	**808 ± 10 nm**	**Total Dose 44 J**	**NR**	**Placebo laser**	**NR**	**Fracture region**	**Sessions:Preoperative and 2, 7, and 14 days after surgery** **Follow-up:Preoperative and 2, 7, and 14 days after surgery**	**1.Pain (VAS)** **2.Mandibular mobility** **3.Facial sensitivity(VAS)** **4.Bite force**
**Nayak, S. S.** **2025**	**India**	**L:16** **P:16**	**L:14/2** **C:12/4**	**L:31.5** **±10.7** **C:33.8** **±10.62**	**Mandibular fractures**	**NR**	**660 and 905 nm combination**	**Total Dose 42.3 J**	**235mW**	**Placebo laser**	**180sec** **per point**	**Fracture region**	**Sessions:After surgery once daily for 4 days**	**1.Pain (a quantitative** **sensory testing algometer)** **2.Mandibular mobility** **3.Facial edema**

Abbreviations: L, Laser group; C, control group; M, Male; F, Female; VAS, Visual analogue scale; NR, Not report; Quick DASH, Quick Questionnaire for Disabilities of the Arm, Shoulder, and Hand; FL, Fracture line; CB, Cortical bridging; PRWE, Patient-Rated Wrist and Hand Evaluation; AROM, Active range of motion; A, Acupuncture group; P, Placebo group; NRS, Numeric Rating Scale; SMI, Sustained Maximal Inspiration; MPQ, McGill Pain Questionnaire.

### Risk of bias and quality of evidence

**Figure 2.**
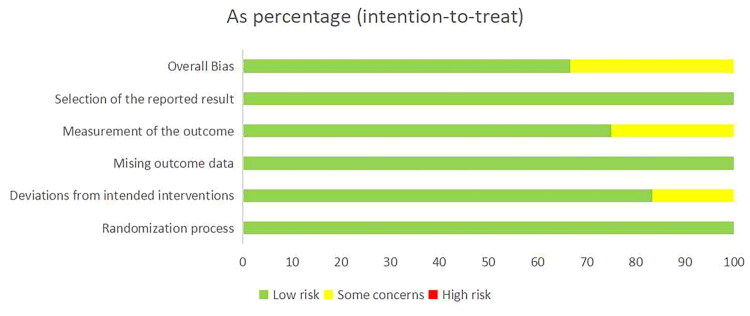
Risk of bias assessment.

**Figure 3.**
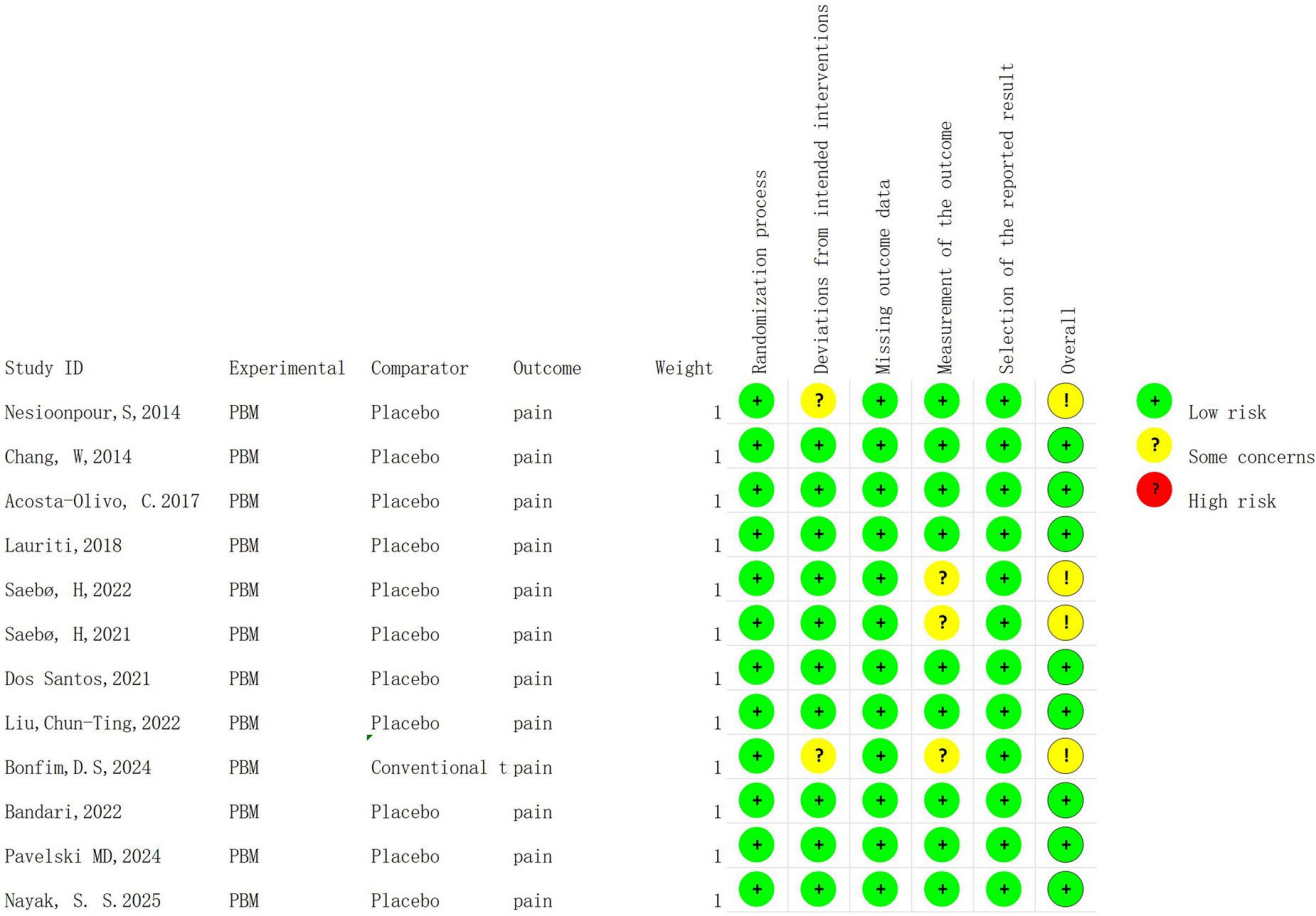
Risk of bias among the included studies.

The assessment of risk of bias is shown in Figure 2. Eight studies showed that all criteria of bias (randomization process, deviation from intended interventions, missing outcome data, outcome measures and choice of reported outcomes) were low risk [24,26–30,34,35]. One study suggests that there are some concerns regarding deviations from intended interventions [31]. One study suggests that there are some concerns regarding deviations from intended interventions and outcome measures [25]. Two studies have some concerns regarding outcome measurements [32,33]. Three studies have patients withdrawn at follow-up, but the probability of patient death or serious consequences caused by the intervention in this trial is extremely low, so the risk of missing outcome data is low (Figure 3) [29,32,33]. According to the GRADE method, due to the risk of bias, the pain score (VAS) aspect was downgraded by 1 point, resulting in the quality of evidence being rated as ‘Moderate’, as shown in Table 3. Owing to the inability to convert some pain scales and the limited number of studies, we did not draw funnel plots.

**Table 3.** Summary of GRADE evidence profile.

Outcome	No of Participants (studies)	Study design	Risk of bias	Inconsistency	Indirectness	Imprecision	Publication bias	Effect size	Quality of the evidence	Importance
								(95% CI)		
Pain score	236(6 studies)	RCT	Serious ^a^	Not Serious	Not Serious	Not Serious	Undetected	SMD −0.72	Moderate	Critical
								(−1.19, −0.24)		
Pain and disability	121(3 studies)	RCT	Serious^a^	Not Serious	Serious ^b^	Not Serious	Undetected	MD −7.3	Low	Important
								(−14.07, −0.54)		
Grip strength	95(2 studies)	RCT	Not Serious	Not Serious	Not Serious	Serious ^c^	Undetected	MD 5.03	Moderate	Important
								(4.29, 5.78)		
Maximum opening	86(3 studies)	RCT	Not Serious	Not Serious	Not Serious	Serious ^c^	Undetected	MD 1	Moderate	Important
								(−0.69, 2.69)		
Analgesic medication	412(2 studies)	RCT	Not Serious	Serious ^e^	Not Serious	Serious ^d^	Undetected	RR 0.66	Low	Not Important
								(0.41 to 1.08)		

Abbreviations: GRADE, quality of evidence grade; CI, confidence interval; RCT, randomized controlled trial;SMD, standardized mean difference; MD, mean difference; RR, risk ratio.

a Some studies have concerns about deviations from intended interventions, incomplete outcomes, and outcome measures.

b The scale only reports the sum of pain scores and disability scores.

c Small number of samples.

d Results are reported as the sum of events at four time points.

e Significantly heterogeneousI^2^=89%.

### Primary outcome

Among the eleven studies included in this systematic review, six studies assessed pain in the short term after surgery *via* the VAS or NRS [24,26,27,29–31], and three studies assessed pain and disability *via* the PRWE [30,32,33]. One study assessed pain in patients with the MPQ scale [25], and two studies had incomplete VAS data [28,34].

**Figure 4.**
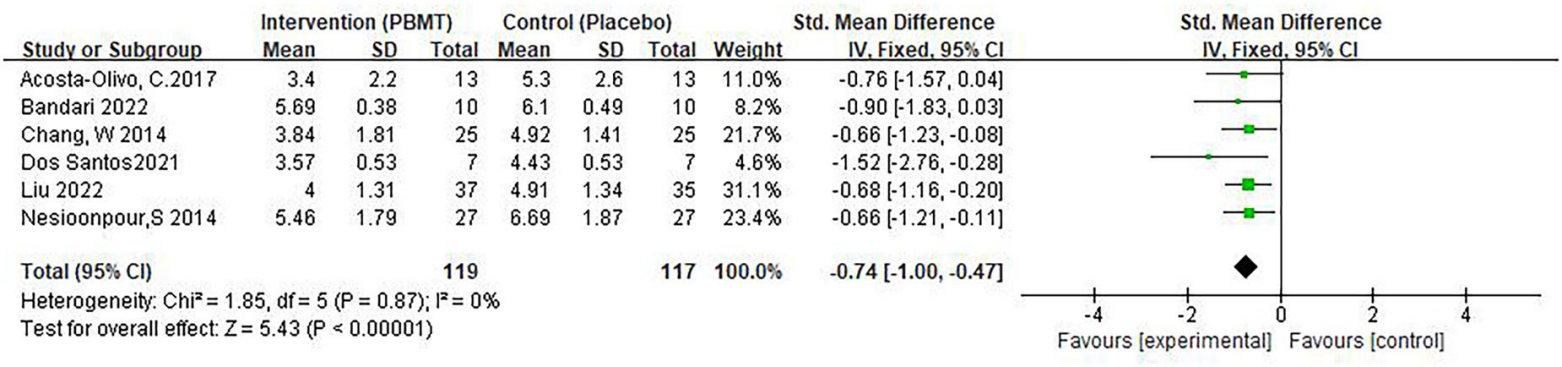
** **A meta-analysis forest plot of pain scores (fixed effects model). The standard mean difference (SMD) and 95% confidence interval (95% CI) are reported. The subjects in the intervention group received photobiomodulation therapy (PBMT), whereas those in the control group received a placebo.

VAS or NRS: Six studies reported scores in the short term after the end of the intervention [24,26,27,29–31]. Due to the variation of studies and the limitation of the number of studies, we comprehensively analyzed the pain scores within one week reported by the included studies. A fixed effects model was applied, with low heterogeneity (I^2^ =0%). Pooled analysis revealed significant differences in pain scores between the two intervention groups (SMD −0.74, 95% CI −1.00, −0.47, *p* < 0.0001; Figure 4).

#### Subgroup analysis

We performed subgroup analysis according to different fracture sites, different irradiation sites, and different control groups.

**Figure 5.**
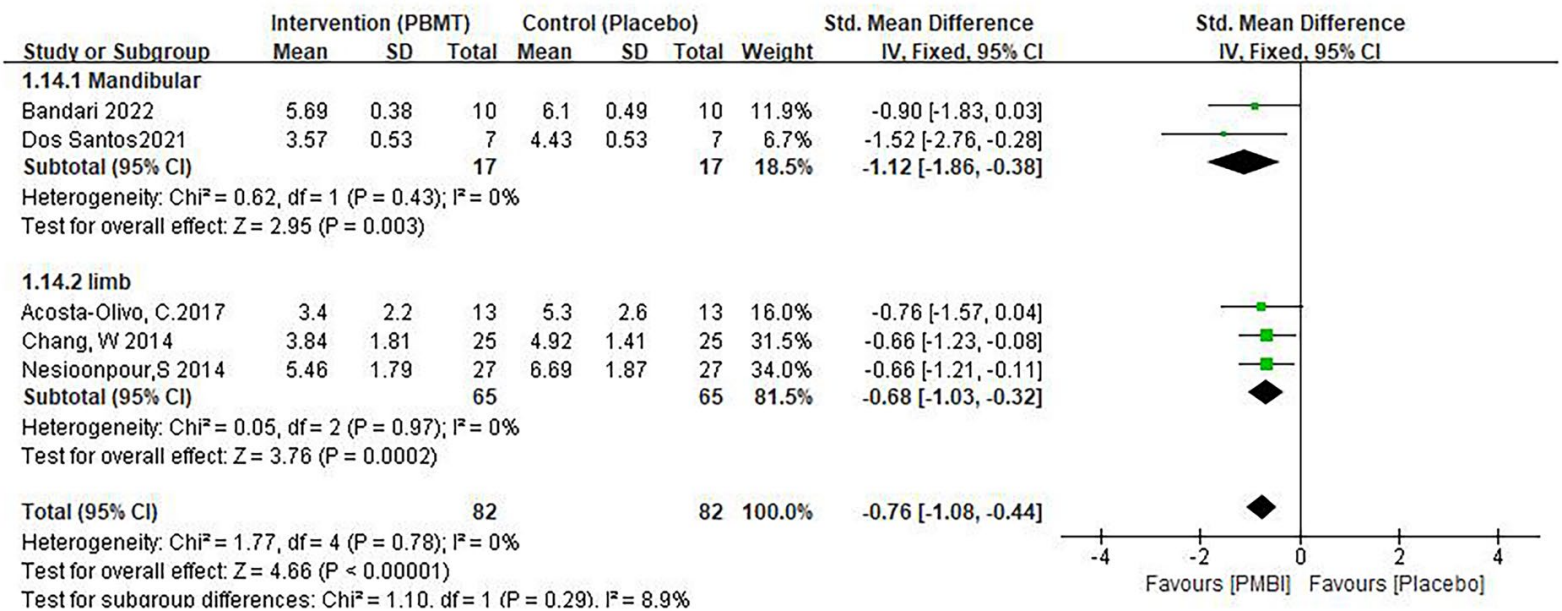
** **A meta-analysis forest plot of the results of the subgroup analysis of pain scores across different fracture sites, including mandibular fractures and limb fractures (fixed effects model). The standard mean difference (SMD) and 95% confidence interval (95% CI) are reported. The subjects in the intervention group received photobiomodulation therapy (PBMT), whereas those in the control group received a placebo.

**Figure 6.**
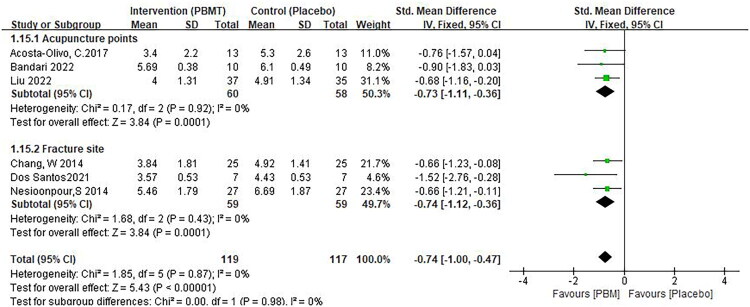
** **A meta-analysis forest plot of the subgroup analysis for pain scores at different irradiation sites, including irradiated acupoints and irradiated fracture sites (fixed effects model). The standard mean difference (SMD) and 95% confidence interval (95% CI) are reported. The subjects in the intervention group received photobiomodulation therapy (PBMT), whereas those in the control group received a placebo.

**Figure 7.**
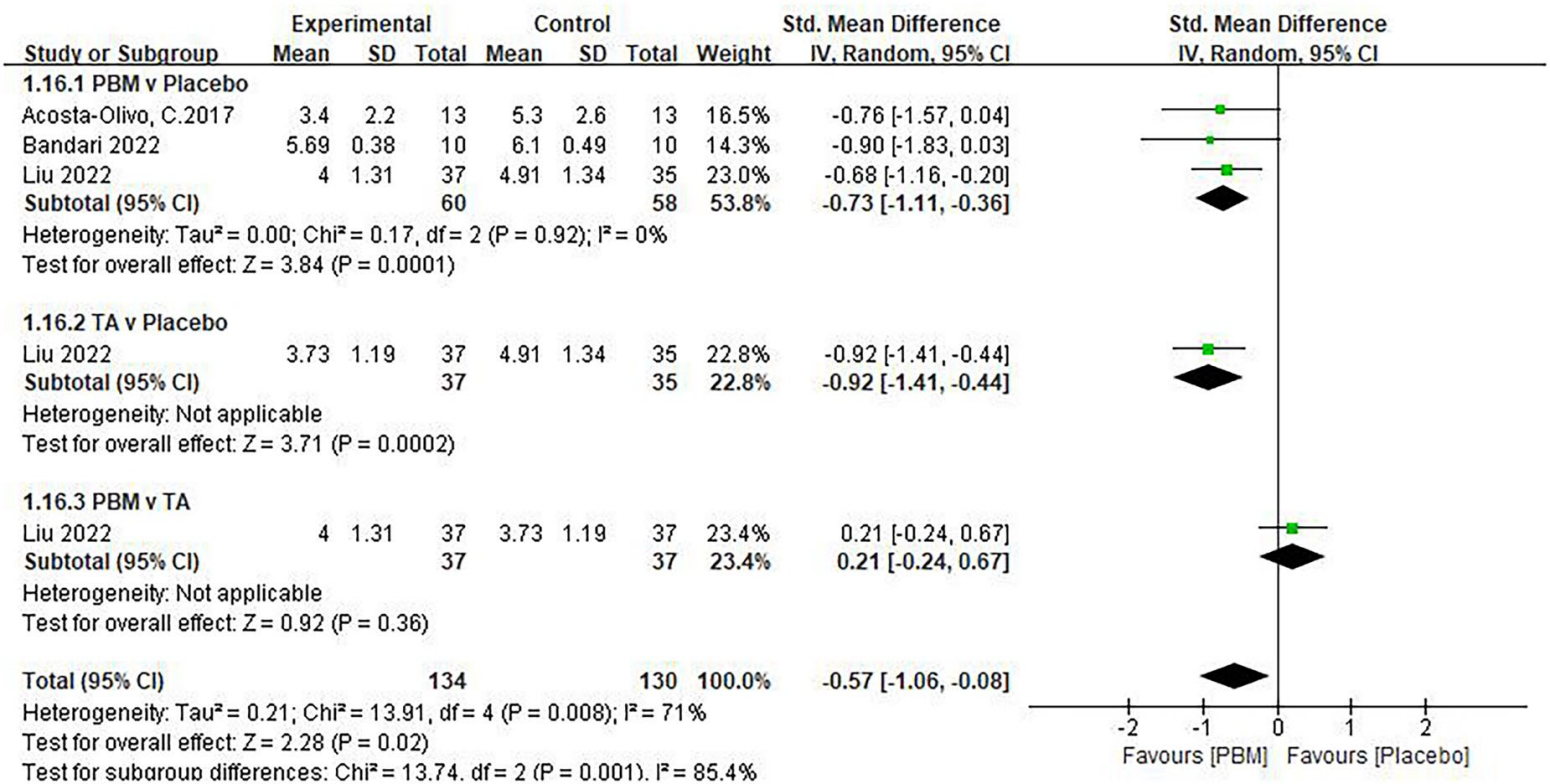
** **A meta-analysis forest plot of the subgroup analysis for pain scores in different control groups (random effects model). The standard mean difference (SMD) and 95% confidence interval (95% CI) are reported. PBM vs. placebo indicates that the subjects in the intervention group received photobiomodulation therapy, and those in the control group received a placebo. TA vs. placebo indicates that the subjects in the intervention group received acupuncture, and those in the control group received a placebo. PBM vs. TA indicates that the subjects in the intervention group received photobiomodulation therapy and those in the control group received acupuncture.

The analgesic effect of PBM in limb fractures and mandibular fractures was statistically significant.(SMD −1.12, 95% CI −1.86, −0.38, *p* = 0.003, Figure 5) [24,27],, (SMD −0.68, 95% CI −1.03, −0.32, *p* < 0.0001, Figure 9) [26,30,31]. The difference between the two groups was statistically significant regardless of irradiation of fracture site or acupoints (SMD −0.73, 95% CI −1.11, −0.36, *p* = 0.01, Figure 6) [24,29,30], (SMD −0.74, 95% CI −1.12, −0.36, *p* < 0.0001, Figure 6) [26,27,31]. In addition, laser irradiation and acupuncture had similar effects on pain relief and were superior to placebo (Figure 7). However, since two of the groups contained only one study, this result should be interpreted with caution.

We supplemented the subgroup analysis according to different wavelength groups and different energy densities.

**Figure 8.**
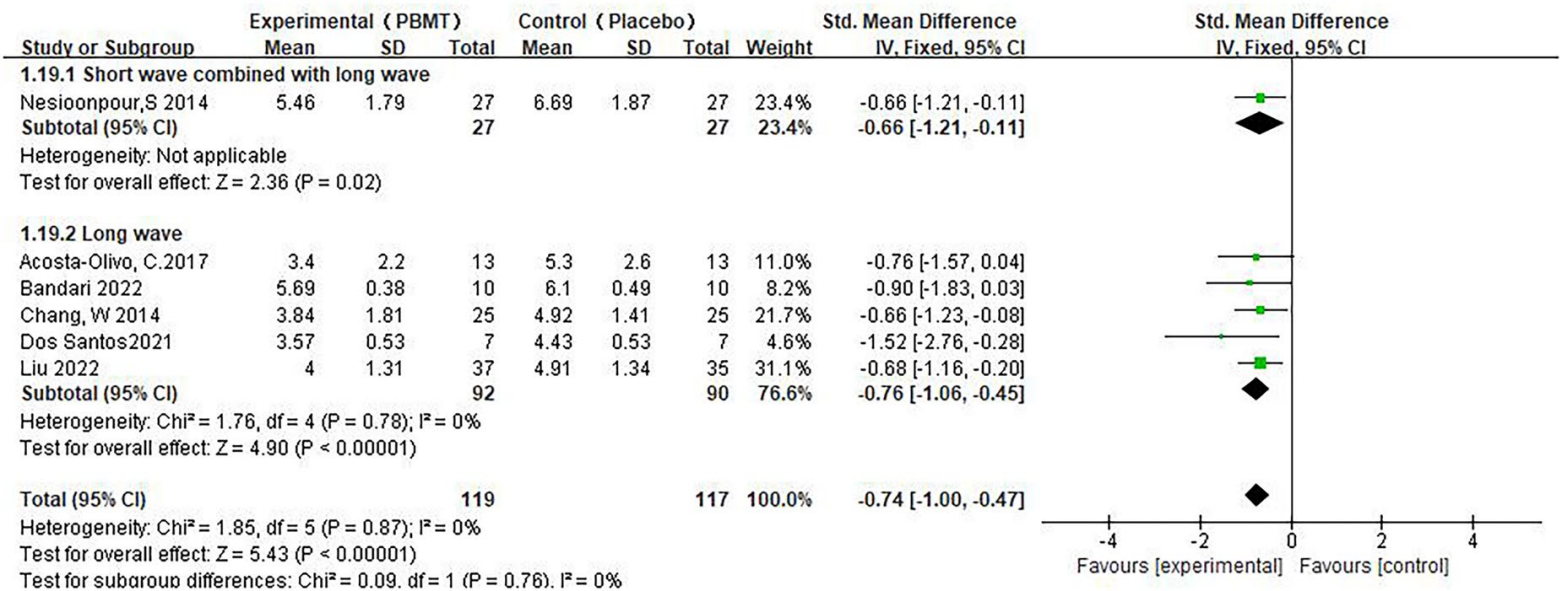
** **A meta-analysis forest plot of the subgroup analysis for pain scores at different wavelengths, including visible red light waves combined with infrared waves and infrared waves only (fixed effects model). The standard mean difference (SMD) and 95% confidence interval (95% CI) are reported. The subjects in the intervention group received photobiomodulation therapy (PBMT), whereas those in the control group received a placebo.

**Figure 9.**
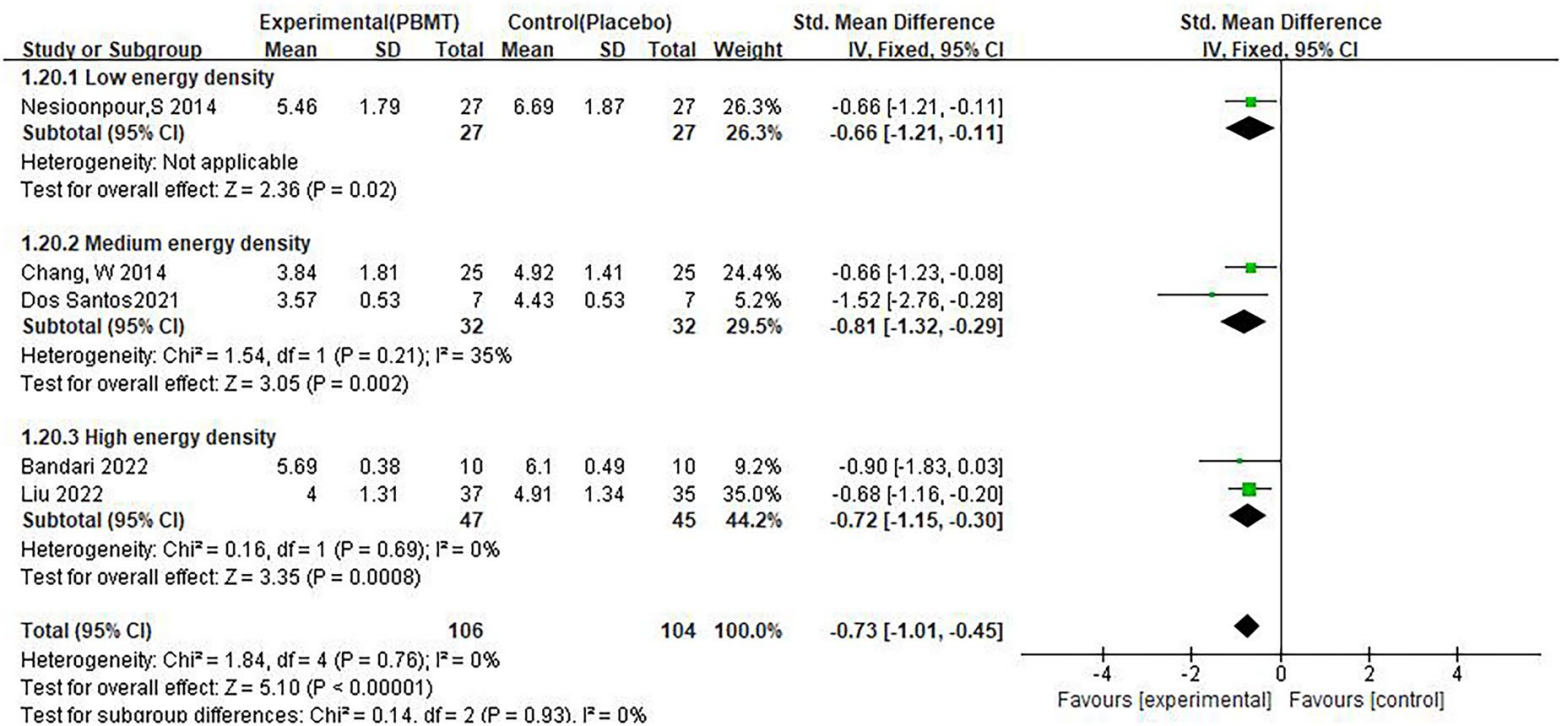
** **A meta-analysis forest plot of the subgroup analysis for pain scores in different energy densities (fixed effects model). The standard mean difference (SMD) and 95% confidence interval (95% CI) are reported. The subjects in the intervention group received photobiomodulation therapy (PBMT), whereas those in the control group received a placebo.

Changes in pain scores were statistically significant in both groups, whether using long waves in combination with short waves or using long waves alone.(SMD −0.74, 95% CI −1.00, −0.47, *p* < 0.01, Figure 8) However, only one study used a combination of long wave and short wave, and no study used short wave alone, so the interpretation of this result should be cautious. We divided the energy density into groups (0.1–5.0, 5.1–10.0, 10.1–15.0 and above) based on the study of de Abreu PTR et al. [36], and the results showed that the changes in pain scores in all three groups were statistically significant(SMD −0.73, 95% CI −1.01, −0.45, *p* < 0.01, Figure 9).

Pavelski MD’s study did not provide specific VAS scores, but only provided a line chart of pain scores over time. The line chart showed that no significant difference between the two groups was observed immediately after surgery; Differences were found at 1 (*p* = 0.011), 2 (*p* = 0.001), 7 (*p* = 0.001), and 14 (*p* = 0.010) days postoperatively, with lower pain scores in the laser group than in the placebo group. Nayak’s study used a quantitative sensory testing algometer(JTECH Medical Commander Echo Algometer, JTECH Medical, USA) to measure pain, and the results showed that the difference between the two groups was not statistically significant. Bonfim’s study used MPQ (McGill Pain Questionnaire) to assess pain, and the results showed that the pain score of the intervention group starting on the ninth day after surgery was significantly lower than that of the placebo group.

### Secondary outcome

**Figure 10.**
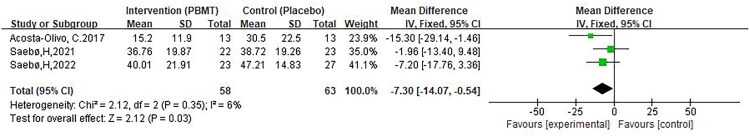
** **A meta-analysis forest plot of pain and disability scores at 4 weeks (fixed effects model). The mean difference (MD) and 95% confidence interval (95% CI) are reported. The subjects in the intervention group received photobiomodulation therapy (PBMT), whereas those in the control group received a placebo.

**Figure 11.**
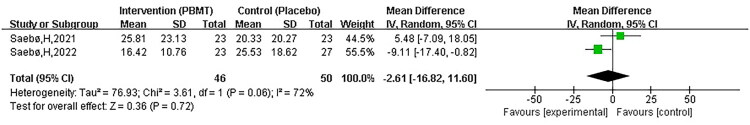
** **A meta-analysis forest plot of pain and disability scores at 8 weeks (random effects model). The mean difference (MD) and 95% confidence interval (95% CI) are reported. The subjects in the intervention group received photobiomodulation therapy (PBMT), whereas those in the control group received a placebo.

**Figure 12.**
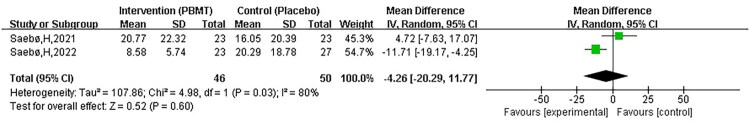
** **A meta-analysis forest plot of pain and disability scores at 12 weeks (random effects model). The mean difference (MD) and 95% confidence interval (95% CI) are reported. The subjects in the intervention group received photobiomodulation therapy (PBMT), whereas those in the control group received a placebo.

**Figure 13.**
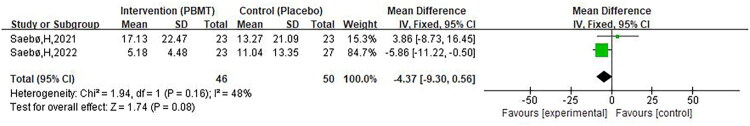
** **A meta-analysis forest plot of pain and disability scores at 26 weeks (fixed effects model). The mean difference (MD) and 95% confidence interval (95% CI) are reported. The subjects in the intervention group received photobiomodulation therapy (PBMT), whereas those in the control group received a placebo.

PRWE: Three studies reported scores at 4 weeks after trauma [30,32,33]. The pooled analysis revealed significant differences between the two groups of interventions (MD −7.30, 95% CI −14.07, −0.54; *p* = 0.04; I^2^ =6%; Figure 10). Forest plot for the pain and disability scores at 4 weeks. Two studies reported scores at 8, 12, and 26 weeks [32,33]. The pooled analysis revealed no significant differences in pain or disability scores at rest between the two groups. There was significant heterogeneity among the studies (Figures 11-13).

#### Functional recovery

**Figure 14.**
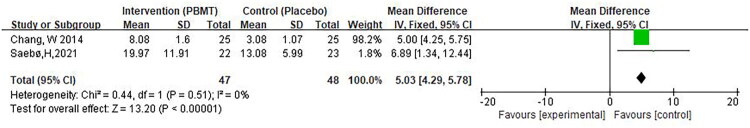
** **A meta-analysis forest plot of grip strength (fixed effects model). The mean difference (MD) and 95% confidence interval (95% CI) are reported. The subjects in the intervention group received photobiomodulation therapy (PBMT), whereas those in the control group received a placebo.

Grip strength: Two studies reported grip strength in patients with upper limb fractures four weeks after trauma [26,32]. Pooled analysis revealed that the grip strength of the intervention group was significantly greater than that of the control group (MD 5.03, 95% CI 4.29, 5.78; *p* < 0.0001, I^2^=0%, Figure 14).

**Figure 15.**
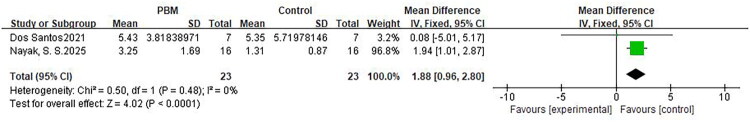
** **A meta-analysis forest plot of the maximum opening distance (fixed effects model). The mean difference (MD) and 95% confidence interval (95% CI) are reported. The subjects in the intervention group received photobiomodulation therapy (PBMT), whereas those in the control group received a placebo.

Maximum opening distance: Two studies reported changes in maximum opening distance in patients with mandibular fractures after one week [27,35]. The pooled analysis showed that the change in maximum opening distance was greater in the intervention group than in the control group (MD 1.88, 95% CI 0.96, 2.80; *p* < 0.0001, Figure 15). A fixed effects model was applied, with low heterogeneity (I^2^=0%). In Pavelski MD’s study, the maximum opening distance was only significantly different during 7 and 14 days, with the laser group being superior to the placebo group, with p-values of 0.0442 and 0.026, respectively. Bandari’s study measured maximum opening distance after one week [24], and Dos Santos tracked maximum opening distance three months after surgery [27]. Neither found a significant difference between the intervention and control groups.

#### Bone healing

**Figure 16.**
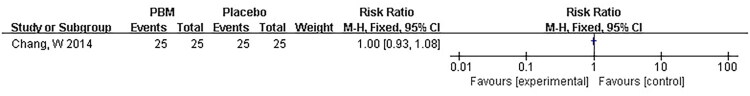
** **A meta-analysis forest plot of observable fracture lines (fixed-effects model). The risk ratio (RR) and 95% confidence interval (95% CI) are reported. The subjects in the intervention group received photobiomodulation therapy (PBMT), whereas those in the control group received a placebo.

**Figure 17.**
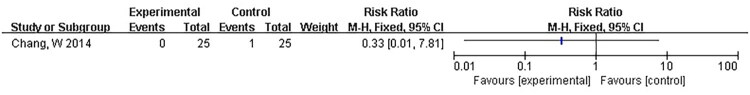
** **A meta-analysis forest plot of the emergence of cortical bridging (fixed-effects model). The risk ratio (RR) and 95% confidence interval (95% CI) are reported. The subjects in the intervention group received photobiomodulation therapy (PBMT), whereas those in the control group received a placebo.

One study used radiographic images to assess whether the fracture line disappeared and whether cortical bridging was detectable (formation of a callus and gradual disappearance of cortical disruption at the fracture site) at two weeks after treatment [26]. Neither analysis revealed a statistically significant difference between the two groups of interventions (Figures 16 and 17). The percentage of bone mineral deposition at days 0, 30, and 60 was recorded by X-ray, and Bonfim, D. S, et al. reported that there was no improvement in bone mineral density in the control group at days 30 and 60[25]. Statistically significant differences were observed in the PBM group at 30 and 60 days (*p* = 0.005 and *p* = 0.002, respectively) compared with day 0.

#### Nocturnal pain

**Figure 18.**
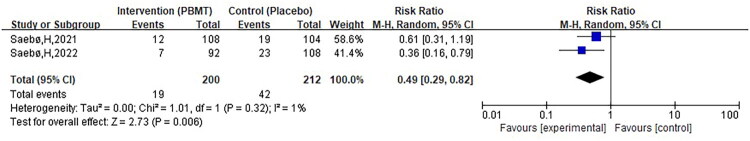
** **A meta-analysis forest plot of nocturnal pain (fixed effects model). The risk ratio (RR) and 95% confidence interval (95% CI) are reported. The subjects in the intervention group received photobiomodulation therapy (PBMT), whereas those in the control group received a placebo.

Two studies reported whether patients experienced nocturnal pain 7–26 weeks after injury (four measurement points) [32,33]. Pooled analysis revealed significant differences in nocturnal pain between the two intervention groups (RR 0.49, 95% CI 0.29, 0.82; *p* = 0.006, I^2^=1%; Figure 18).

#### Use of analgesic drugs

**Figure 19.**
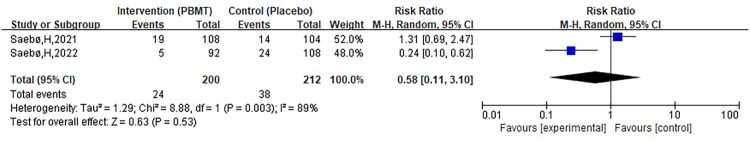
** **A meta-analysis forest plot of analgesic drugs (fixed effects model). The risk ratio (RR) and 95% confidence interval (95% CI) are reported. The subjects in the intervention group received photobiomodulation therapy (PBMT), whereas those in the control group received a placebo.

Two studies reported whether patients used analgesic drugs 7–26 weeks after injury (four measurement points) [32,33]. The pooled analysis revealed no significant differences in the use of analgesic drugs between the two intervention groups (RR 0.58, 95% CI 0.11, 3.10; *p* = 0.53, I^2^=89%; Figure 19).

#### Sensitivity analysis

For pain, by sequentially excluding each study to assess the robustness of the results, sensitivity analyses showed that no individual study data affected the combined effect estimate. No sensitivity analysis was performed due to the small number of other outcome studies. The number of studies reporting the primary outcome measure was small, so no funnel plot was drawn.

## Discussion

This systematic review discussed the efficacy of PBM in patients with fractures, including pain, function, and bone healing. A previous systematic review by Neto FCJ et al. explored the efficacy of PBM in patients with fractures [27]. However, the small number of included studies and the low quality of evidence led to increased uncertainty in the estimated effect values. The accumulated evidence of our study confirms and reinforces the previous findings that, compared with placebo or conventional treatment, PBM can reduce short-term postoperative pain in patients with fractures, with no evidence to support its long-term efficacy. We additionally found that PBM may promote grip strength recovery in patients with upper limb fractures, and mandibular functional rehabilitation remains controversial, but subgroup analyses showed no significant differences in pain relief between the two. In addition, the subgroup analysis also found that PBM therapy and laser acupuncture had similar analgesic effects. The wavelengths involved in the included studies did not differ in the efficacy of pain relief. There is no clear evidence that PBM can promote bone healing. No side effects were reported in the included studies. These are primarily based on low-to-moderate quality evidence.

Pain control is beneficial for patient recovery, and the repair of damaged tissue can alleviate patient pain. Therefore, pain management and rehabilitation are mutually reinforcing processes.To accelerate the recovery process of fracture patients and improve their comfort and treatment compliance, we should pay attention to their pain management. The management of post-fracture pain is an important topic in clinical practice, and its complexity stems from the multiple mechanisms of pain: periosteal irritation, soft tissue damage, inflammatory response, muscle spasm and possibly nerve damage together form the pathological basis of fracture pain. In this context, it is often difficult to achieve the ideal analgesic effect by relying on one analgesic method alone, and may be limited by adverse drug reactions. Multimodal analgesia achieves the goal of synergistic analgesia, reducing drug dosage, and reducing adverse reactions by combining drugs with different mechanisms of action (such as NSAIDs, acetaminophen, opioids, etc.) and non-pharmacological interventions (such as cold therapy, nerve block, rehabilitation training, etc.) [37]. The Guidelines for Accelerated Rehabilitation Surgery (Enhanced Recovery After Surgery) also highlight that multimodal analgesia is one of the core strategies to optimize perioperative management and promote early functional recovery [38]. Within this framework, photobiomodulatory therapy (PBM) has a unique adjunctive value.

Our study revealed that PBM appears to be associated with early postoperative pain reduction in patients with fractures (1 weeks), which is consistent with the findings of Neto FCJ [27]. Only two studies tracked patients’ long-term pain and functional scores and found no effects(4 to 26 weeks). We tried to find the reasons for this outcome, but we did not find a source of heterogeneity in the study design, treatment options, and the course of rehabilitation training. This temporal pattern can be explained by two mutually non-exclusive mechanisms. First, PBM is mainly targeted at acute inflammation and early repair stages, and its effect is diminished once the fracture enters the remodeling stage. Although there are currently no other systematic reviews exploring the efficacy of PBM in patients with fractures, numerous systematic reviews of animal and human trials have suggested that PBM promotes tissue healing and has anti-inflammatory and analgesic effects on muscle and skeletal diseases [14,17,18,39,40]. Notably, patients with different recovery statuses may report similar pain levels at rest but differ substantially in pain during movement or joint activity. Therefore, the future evaluation indicators for long-term fracture efficacy should focus on operative pain and function.

Due to the small number of included studies and sample size, there is some controversy regarding the effect of PBM on mandibular function. A combined analysis of the two studies suggests that PBM can increase the maximum open distance of mandibular fractures, but the two studies that could not be included in the meta-analysis did not find the efficacy of PBM in this respect. No significant heterogeneity and low quality studies were found. Our findings suggest that PBM may promote grip strength recovery in patients with upper limb fractures to some extent, but does not show advantages for the evaluation of overall function. The reason for this result may be that due to different anatomical structures and fracture types, both of them are affected by many factors in the rehabilitation process, and this meta-analysis involves fewer patients with mandibular fractures. At present, there are no studies directly comparing the differences in the rehabilitation process of different fracture sites. Due to the limited number of studies, we were unable to obtain additional information to assess the effects of long-term interventions. Although no studies have directly evaluated the effect of PBM on the function of fracture patients, some studies have shown that lasers can promote the recovery of muscles and nerves [23,41,42]. Fractures can also damage muscles and tissues near the fracture site. There is a theoretical possibility that PBM can promote the function of fracture patients. Therefore, the effectiveness of PBM in the functional recovery of fracture patients still needs more trials for verification.

In addition, this study found that laser irradiation at both acupoints and fracture sites can reduce the pain of patients. There is no systematic review comparing the effects of low-level laser irradiation on wounds and acupoints. The two different uses of the PBM focus on the two mechanisms of PMBT. When low-level lasers irradiate wounds, they can inhibit the inflammatory response, promote the activation of growth factors, and accelerate tissue repair [15,16,43]. In addition to stimulating the photobiological stimulation effect, laser acupuncture can also stimulate acupuncture points painlessly to achieve effects similar to those of acupuncture [44]. A meta-analysis revealed that [45], laser acupuncture was able to significantly reduce instantaneous pain levels and improve instantaneous mouth opening ability in patients with mandibular joint disorders. Moderate-quality evidence by Law D et al. suggests that appropriate doses of laser acupuncture are able to improve musculoskeletal pain [46].

Subgroup analysis also showed that acupuncture and laser acupuncture had similar effects on relieving the pain of fracture patients and were better than the placebo. Acupuncture originated in China 4,000 years ago, and there are many hypotheses about its mechanism, but none of them can provide a complete explanation. Its main mechanisms include the following aspects: 1) unblocking meridians and promoting qi and blood flow (TCM theory) [47]; 2) activating endogenous opioid peptides (e.g. endorphins) for analgesia [48]; 3) modulating neurotransmitters like serotonin and norepinephrine [49]; 4) inhibiting inflammatory factors [50]; 5) blocking pain signal transmission in the spinal cord [51]; 6) diffuse noxious inhibitory control [52,53]; 7) improving local blood circulation [53]; 8) possible psychological effects (e.g. distraction, positive expectations) [48,52]. But there are fewer studies and sample sizes involved in this. More research is likewise needed to compare the differences in efficacy and safety between laser acupuncture and acupuncture.

Lopes et al. reported that PBM therapy is capable of improving bone healing in dentistry [19]. A systematic review of animal experiments revealed that PBM can promote the healing of bone defects [39]. In addition to increasing ATP production, promoting growth factor secretion and stimulating osteocyte proliferation, PBM can also increase vascularization and reduce the inflammatory response to create a favorable environment for bone healing. In this review, only two studies evaluated bone healing in patients with fractures [54]. One of the studies documented whether the fracture line disappeared and whether cortical bridging was detectable at two weeks. No significant changes were found. Another study revealed that the percentage of bone mineral deposition in the PBM group was greater than that in the control group at 30 and 60 days after the intervention, and the difference was significant. Different recording times and indicators contribute to this difference in results. With the continuous deposition of bone minerals, the bone tissue at the fracture end will be connected together and gradually become harder. Bone mineral deposition and cortical bridging are different important links in the process of fracture healing, and the disappearance of fracture line is the sign of complete fracture healing. Only when bone minerals are deposited to a certain extent can changes be observed radiographically. The results of this study are insufficient to support the efficacy of PBM in promoting bone healing in fracture patients. Therefore, more studies are needed to verify the effectiveness of PBM for bone healing using the same or similar assessment modalities.

Photobiomodulation (PBM), also known as low-level lasers, is a noninvasive and nondrug therapy. The light emitted by PBM is absorbed by cytochrome C oxidase of the mitochondrial respiratory chain, stimulating cellular metabolism and regulating cellular function [10,13]. These photophysical and photochemical reactions can increase ATP production, provide energy to cells, regulate immune cells to inhibit inflammation [15,16,55], and promote the activation of growth factors (platelet-derived growth factor, fibroblast growth factor) to accelerate tissue regeneration [43,55]. In addition, PBM can affect nerve cells. It can stimulate nerve cells to release endogenous analgesic substances (such as β-endorphins), increase the release of inhibitory neurotransmitters or reduce the release of excitatory neurotransmitters [56], inhibit the activation of nociceptors and the transmission of pain signals, promote nerve regeneration and repair, etc [57]. Therefore, PBM may play a positive role in promoting wound healing and reducing pain. However, the mechanism of PBM may vary depending on treatment parameters (wavelength, power, etc.) and individual differences. Photobiomodulation mainly occurs in the near-infrared and visible bands of the electromagnetic spectrum, and the wavelength range is usually 400–950 nm. Among them, the red light band (630–660 nm) and near-infrared light band (810–830 nm) are widely used because of their good tissue penetration ability and biological effects [43]. Different wavelengths and irradiation time will affect the efficacy of PBMT. In our study, except for the experiment of Nesioonpour S, which is short wave combined with long wave, the rest of the wavelengths are between 780 and 1300 nm. No studies used visible red light alone, and the results of this subgroup analysis should be interpreted with caution. Visible red light (600–700 nm) is mainly absorbed by superficial tissues and is suitable for epidermal or superficial lesions [58]; While near-infrared light (780–1300 nm) penetrates deeper and can act on deep tissues such as bones, joints and muscles [59]. Available evidence mainly supports the use of near-infrared light in fracture-related pain management. According to the results of Nesioonpour S, we cannot deny the efficacy of visible red light on fractures. Fracture patients are usually accompanied by trauma. The combination of the two wavelengths may promote the recovery of superficial epidermal tissue and deep skeletal muscle tissue at the same time, but there is no clear evidence to confirm this. It is necessary for future studies to directly compare the efficacy differences of different wavelengths in the same fracture model to clarify the optimal wavelength choice.

Because the irradiation duration and power density were highly mixed in the original study, the subgroup analysis based on irradiation duration alone lacked biological significance. Therefore, according to the method proposed by de Abreu et al. this study divided the energy density into three levels (0.1–5.0 J/cm^2^, 5.1–10.0 J/cm^2^, 10.1–15.0 J/cm^2^ and above) for subgroup analysis, representing low, medium and high doses, respectively. The results of the analysis showed that all dose groups showed a statistically significant pain relief effect. This finding suggests that the relief effect of PBM on post-fracture pain is relatively stable within the energy density ranges currently employed in the included studies, with no significant dose-dependent differences observed. However, this result needs to be interpreted carefully. The calculation of energy density depends on the accurate report of power density and spot area in the original study. Some studies have incomplete parameter reports, which may introduce calculation errors. Some studies have suggested that PBMT may have a dose-dependent problem. Within the treatment window, too low exposure amplitude is ineffective, while too high exposure may have an inhibitory effect [60]. One study employed an energy density of up to 100 J/cm^2^, but still showed efficacy in subgroup analysis, which may suggest that different tissues (such as bone and soft tissue) have differences in sensitivity to light dose, or that high doses still have therapeutic value in some clinical scenarios. At present, there is no authoritative study that explicitly suggests the best treatment window for PBM to promote bone healing. It is recommended that follow-up studies explicitly report the power density, spot area and irradiation time, so as to more accurately analyze the dose-response relationship.

Few trials have reported nocturnal pain and analgesic consumption in detail. Heidari M et al. noted that PBM can reduce nocturnal pain after dental surgery and the consumption of analgesics [6]. Our study revealed that less nocturnal pain occurred in the PBM group and that there was no significant difference in analgesic medication consumption. These two outcomes are only used to assist in evaluating the analgesic effect of PBM and the quality of evidence is low.

## Study limitations

For the PRWE scale, we were unable to obtain scores for its pain and functional components separately, so we analyzed only the total score. The number of trials evaluating the medium-and long-term efficacy of PBM in fracture patients is small, and more high-quality studies evaluating the long-term efficacy of PBM are needed in the future. The number of tests to evaluate bone healing is small, and the evaluation methods are not uniform. We also need more imaging findings to assess the actual healing of fracture patients. In addition, the efficacy of PBM depends on instrument parameters (wavelength, frequency of irradiation, etc.). However, existing studies do not support our subgroup analysis based on wavelength and irradiation frequency. In the future, more trials are needed to determine the optimal laser parameters for patients with fractures. The difference in efficacy and safety between laser acupuncture and acupuncture also needs more exploration. While no studies have reported side effects or adverse reactions, new studies are needed to evaluate the safety of therapeutic band low-level lasers. Finally, only a few studies have evaluated the functional recovery of patients and only involved upper extremity fractures and mandible fractures. Notably, none of the included studies evaluated angiogenesis-related outcomes. Given that angiogenesis plays a key role in bone remodeling and is considered a key mechanism for photobiomodulating therapeutic effects. Future studies should incorporate angiogenesis endpoints to better elucidate the mechanism by which PBM promotes fracture healing. It is also regrettable that publication bias cannot be evaluated.

## Conclusion

Based on low-to-moderate quality evidence from 12 randomized controlled trials (9 in the meta-analysis), PBM appears to reduce short-term pain in patients with fractures (at 1 week) and promote the recovery of grip strength in upper limb fractures (at 4 weeks), but there is no clear evidence of promoting bone healing. Subgroup analysis showed that PBM was effective whether irradiating the fracture site or adjacent acupuncture points, and laser acupuncture had similar analgesic effects as traditional acupuncture. Pain relief was observed in different energy density and wavelength protocols (780 ∼ 1300 nm). However, no significant long-term benefit was observed for pain or function. Evidence for mandibular function recovery remains inconsistent. The PBM group showed fewer nocturnal pain and no difference in analgesic consumption. No adverse reactions were reported. Future trials should optimize device parameters, extend the duration of follow-up for exercise-induced pain and functional outcomes, determine the measures of bone healing, incorporate angiogenesis endpoints, and systematically evaluate safety.

## Supplementary Material

Clean Manuscript.docx

IANN]_OpenScienceForm.docx

ROB 2.png

## Data Availability

This was a systematic review and meta-analysis with data from previous studies. The data ultimately produced by the study will be obtained from the project leader for reasonable reasons.

## Open scholarship

This article has earned the Center for Open Science badges for Preregistered. The data and materials are openly accessible at https://www.crd.york.ac.uk/PROSPERO/view/CRD42024591373.
